# Reversal of the Caspase-Dependent Apoptotic Cytotoxicity Pathway by Taurine from *Lycium barbarum* (Goji Berry) in Human Retinal Pigment Epithelial Cells: Potential Benefit in Diabetic Retinopathy

**DOI:** 10.1155/2012/323784

**Published:** 2012-04-11

**Authors:** M. K. Song, B. D. Roufogalis, T. H. W. Huang

**Affiliations:** Herbal Medicine Group, Faculty of Pharmacy, The University of Sydney, Sydney, NSW 2006, Australia

## Abstract

Diabetic retinopathy is a preventable microvascular diabetic complication and a leading cause of vision loss. Retinal pigment epithelial cell apoptosis is an early event in diabetic retinopathy. Taurine is reportedly beneficial for diabetic retinopathy and is abundant in the fruit of *Lycium barbarum* (LB). We have investigated the effect of pure taurine and an extract of LB rich in taurine on a model of diabetic retinopathy, the retinal ARPE-19 cell line exposed to high glucose. We demonstrate for the first time that LB extract and the active ligand, taurine, dose dependently enhance cell viability following high glucose treatment in the ARPE-19 retinal epithelial cell line. This cytoprotective effect was associated with the attenuation of high glucose-induced apoptosis, which was shown by characteristic morphological staining and the dose-dependent decrease in the number of apoptotic cells, determined by flow cytometry. Moreover, we have shown that LB extract and taurine dose dependently downregulate caspase-3 protein expression and the enzymatic activity of caspase-3. We conclude that taurine, a major component of LB, and the LB extract, have a cytoprotective effect against glucose exposure in a human retinal epithelial cell line and may provide useful approaches to delaying diabetic retinopathy progression.

## 1. Introduction

Diabetic retinopathy (DR) is one of the most common microvascular complications of diabetes and remains a major cause of preventable blindness worldwide [1]. Diabetes damages all the major cells of the retina and pigment epithelial cells [2]. This results in increased blood flow and capillary diameter, proliferation of the extracellular matrix and thickening of basal membranes, altered cell turnover (apoptosis, proliferation), and breakdown of the blood retinal barrier [3].

Apoptosis or programmed cell death can be triggered by various signals and is characterized by well-defined morphologic changes, including chromatin condensation and fragmentation, and formation of apoptotic bodies [4]. Retinal microvascular cells are lost selectively via apoptosis before other histopathology is detectable in diabetes [5]. Moreover, recent findings have suggested that apoptotic episodes in retinal cells during the initial stage of diabetes play an integral role in the early stage of vision loss [6]. Therefore, apoptosis is important in the progression and pathogenesis of DR [7].

Retinal pigment epithelial (RPE) cells form a monolayer between the neuroretina and the choriocapillaris, which are the essential components of the outer blood retinal barrier (BRB) that maintains physiological and structural balance within the retina [8]. RPE cells are particularly susceptible to oxidative stress because of high oxygen consumption by photoreceptors [9]. Moreover, recent studies have shown that RPE cells undergo oxidative stress and UV-light induced apoptosis [10, 11]. Although several studies have shown that RPE degenerates in the early stage of diabetes [12, 13], the mechanisms of high-glucose-induced apoptosis in RPE cell models of DR are not fully understood.

High glucose causes activation of several proteins involved in apoptotic cell death, including members of the caspase family [14]. It is well known that caspases are involved in the initiation and execution of apoptosis [15]. Moreover, the widely studied caspase-3 plays an important role in diabetes [16].

Recent studies have suggested that ligand-activated PPAR-*γ* controls apoptosis, contributing to tissue protection [17]. Indeed, it has been shown that the PPAR-*γ* agonist rosiglitazone protects against oxidative stress-induced apoptosis through upregulation of antiapoptotic Bcl*-*2 family proteins [18]. Troglitazone showed a cytoprotective activity from oxidative stress-induced apoptosis in ARPE-19 cells [19]. Moreover, the study has shown that a natural ligand, 15-deoxy-delta-12,14-prostaglandin J_2_ (15d-PGJ_2_), helps RPE cells to maintain mitochondrial integrity by prevention of cytochrome c (cyt c) release from mitochondria and subsequent activation of the apoptosis pathway [20].

Fruits from *Lycium barbarum L.* (LB) in the family Solanaceae are a well-known traditional Chinese medicine which has glucose-lowering activity and antiapoptotic activity [21]. There is a growing body of evidence indicating that LB intake increases the fasting plasma zeaxanthin levels, beneficial for maintaining macular pigment density in age-related macular degeneration [22]. Moreover, polysaccharides of LB (LBP) increase antiapoptotic protein Bcl-2 levels in lens epithelial cells [23]. LB contains 18 types of amino acids, including taurine, a nonessential free amino acid, which is one of the chemical components most abundant in LB [24, 25]. Taurine has been recommended as a complementary therapeutic agent for the prevention of diabetic complications in type II diabetes [26]. Moreover, it has been shown that taurine inhibits the activation of caspase-3 in ischemic cardiomyocytes [27]. Taurine has also been found to prevent high-glucose-mediated endothelial cell apoptosis through its antioxidant property [28]. Recently, we have shown that LB extract and pure taurine, a major component in the LB extract, activated PPAR-*γ* by luciferase reporter gene analysis and by mRNA and western blotting measurement in human retinal pigment epithelial cells. At the same time, both LB extract and pure taurine inhibited a variety of PPAR-*γ*-dependent downstream effectors in the retinal cells [29]. However, the pathways of the beneficial effect of LB and its taurine active component on DR by modulation of high-glucose-induced apoptosis through the PPAR-*γ*-mediated caspase-3 pathway have not been investigated. Therefore, the aim of this study is to investigate the cytoprotective effect of pure taurine and an extract of LB rich in taurine against glucose exposure in a human retinal epithelial cell line as a model of DR.

## 2. Materials and Methods

### 2.1. Preparation of LB Extract

Dried LB was purchased in raw powder form (batch no. 53101: DeDu Holdings, West Ryde, Australia). The preparation of an extract of LB was performed as previously described [29, 30] with modifications. Briefly, 250 mg of fine LB powder was extracted with 2.5 mL of methanol by sonication at room temperature for 15 min followed by centrifugation for 5 min. This step was repeated four times. The solvent was then evaporated under reduced pressure below 50°C, and the remaining solid was collected. The identification and quantification of taurine in the LB extract were undertaken by thin layer chromatography (TLC) analysis as previously described [29].

### 2.2. Tissue Culture and Treatment

The human retinal epithelial cell line, ARPE-19, was provided by Dr. Weiyong Shen (Save Sight Institute, Sydney, Australia). Cells were cultured as previously described [31]. Briefly, the cells were cultured in a humidified incubator at 37°C in 5% CO_2_ in 10% fetal bovine serum-defined minimal essential medium (FBS-DMEM)-F12 medium) containing 5.5 mM D-glucose, supplemented with 100 U/mL penicillin G and 100 *μ*g/mL streptomycin. The culture medium was replaced with fresh medium every second day. Upon confluence, cultures were passaged by dissociation in 0.05% (w/v) trypsin (Gibco-Life Technologies, Roseville, MD, USA) in phosphate-buffered saline (PBS) pH 7.4. For high-glucose-induced functional studies, cells were maintained in fresh medium containing 1% FBS for 2 h prior to use in the experiments. Cells were then pretreated with samples, rosiglitazone (RG), 15-deoxy-delta (12, 14)-prostaglandin J_2_ (PG), or vehicle (0.5% DMSO) for 6 h followed by further exposure to normal (5.5 mM) or high (33.3 mM) D-glucose for 48 h. Cells incubated in 27.5 mM mannitol (M) served as osmotic control [32].

### 2.3. Cell Viability by MTS Assay

Cell proliferation was assessed by 3-(4,5-dimethylthiazol-2-yl)-5-(3-carboxymethoxy phenyl)-2-(4-sulfophenyl)-2H-tetrazolium (MTS) assay using the Cell Titer 96 Aqueous One Solution Cell Proliferation Assay Kit (Promega, Madison, WI, USA), as previously described with some modifications [33]. Briefly, ARPE-19 cells were cultured in 96-well plates (3 × 10^4^/mL). Upon confluence, cells were treated with vehicle (0.05% DMSO), RG, colchicine (Colch), or samples for 24 h, 48 h, and 72 h. For high-glucose cytotoxicity experiments, the medium was replaced with fresh medium containing 1% FBS for 2 h prior to use in the experiments. Cells were then preincubated with treatment samples, RG, PG, or vehicle (0.5% DMSO) for 6 h followed by exposure to normal (5.5 mM) or high (33.3 mM) D-glucose for a further 48 h. On the day of the proliferation assay, 20 *μ*L of the MTS solution was added to each of the 96 wells and incubated at 37°C for 1 h in a humidified (5% CO_2_) environment. The absorbance at 490 nm was read in a microplate reader (Bio-Rad Laboratories, Inc, CA). The percentage of cell proliferation was calculated as (OD of treated samples/OD of untreated control) × 100.

### 2.4. Detection of Apoptotic Cells

Cells undergoing apoptosis were determined by Hoechst 33342 staining and Annexin V flow cytometry analysis, as previously described with modifications [4]. Briefly, cells were fixed in 4% formaldehyde for 10 min and stained with Hoechst dye for 10 min. Cells were visualized under an inverted fluorescence microscope (excitation at 365 nm and emission at 480 nm, using a UV filter). A minimum of 300 cells in each cover slip, from six randomly selected fields, were counted, and apoptotic cells were expressed as percentage of total cells counts.

### 2.5. Quantification of Apoptotic Cells

Apoptotic cells were quantified by flow cytometry using FITC Annexin V apoptosis detection kit (BD Biosciences, San Jose, USA), as previously described [34]. Briefly, after appropriate treatments, 1 × 10^5^ cells/100 *μ*L were collected, and 5 *μ*L of FITC Annexin V and 5 *μ*L of propidium iodide (PI) were added. Cells were incubated in the dark at 25°C. After 15 min, 400 *μ*L of 1x binding buffer was added to the cells. The Annexin V-positive (+)/PI-negative (−) cells, indicating early apoptotic cells, and the Annexin V-positive (+)/PI-positive (+), indicating late apoptotic cells, were detected by FACSCalibur (BD Biosciences, San Jose, USA). The results were analysed using the WINDI 2.5 software. Annexin V-FITC conjugates were detected at the FL1 channel, and PI was read on the FL3 channel.

### 2.6. Caspase-3 Activity Assay

Caspase-3 activity was measured by the caspase-3 fluorometric assay system (BD Biosciences, San Jose, USA), as previously described [35] with modifications. Briefly, a total of 1 × 10^6^ cells were harvested and resuspended in cold cell lysis buffer (10 mM Tris-HCL, 10 mM NaH_2_PO_4_/NaHPO_4_ (pH7.5), 130 mM NaCl, 1% Triton-X-100, and 10 mM sodium pyrophosphate (NaPPi)) for 30 min on ice. Cell lysate protein concentrations were determined by the BCA (bicinchoninic acid) assay (Thermo, USA) according to the manufacturer's instructions. Equal amounts of protein were added to each well in a 96-well plate containing 200 *μ*L of HEPES buffer and 5 *μ*L of the fluorogenic substrate, DEVD-AMC (7-amino-4-methylcoumarin), and incubated for 1 h at 37°C. Cleavage of the substrate by active caspase-3 resulted in an increase of fluorescence (excitation = 380 nm and emission = 460 nm), measured in a 96-well plate fluorometer (FLUOstar OPTIMA), and expressed as unit per milligram protein.

### 2.7. Protein Extraction and Semiquantitative Western Blotting Analysis

Immunoblots were conducted as described previously [36]. The proteins from the cells were prepared using the Ripa lysis buffer (25 mM Tris buffer (pH 7.6), 150 mM NaCl, 1% NP-40, 1% sodium deoxycholate, and 0.1% SDS). The lysed cells were centrifuged at 12,000 rpm (Micromax RF centrifuge, Thermo IEC, MA, USA) for 10 min and supernatants resolved by SDS-PAGE, 4–12% (Invitrogen, Australia). Protein was transferred to cellulose membrane in transfer buffer (Tris base 25 mM, glycine 192 mM, pH 8.3) and blocked in 5% skim milk powder (Sigma-Aldrich, St. Louis, MO, USA) overnight. The primary antibodies (Santa Cruz Biotechnology, USA) were anticaspase-3 rabbit polyclonal primary antibodies (1 : 500 dilution). After incubation with the primary antibody for 1 hr at room temperature, the membrane was washed and further incubated with horseradish peroxidase-conjugated anti-mouse secondary antibodies (1 : 6000 dilution; Santa Cruz Biotechnology, USA). Bound antibodies were detected using enhanced chemiluminescence with Lumi-Light Western Blotting Substrate (Roche). The membranes were exposed to X-ray film (Kodak, USA) and developed using the SRX-101A X-ray developer (Konica, Taiwan). The resultant films were quantified by scanning densitometry using ImageJ (National Institutes of Health, Bethesda, MD). Protein expression was quantified by normalization to *α*-tubulin. The membranes were reprobed with anti-*α*-tubulin primary antibody (1 : 10,000 dilution; Santa Cruz Biotechnology, USA) after stripping and overnight blotting with 5% skim milk. The membranes were reincubated with horseradish peroxidase-conjugated anti-mouse secondary antibody and detected using the same procedure as described above. Cell lysate protein concentrations were determined by the BCA (bicinchoninic acid) assay (Thermo, USA) according to the manufacturer's instructions.

### 2.8. Chemicals

Rosiglitazone (RG) was purchased from Alexis Biochemicals (San Diego, CA, USA). Pure taurine compound, 15-deoxy-delta (12, 14)-prostaglandin J_2_(PG), and other chemicals were purchased from Sigma-Aldrich (St. Louis, MO, USA), unless otherwise indicated.

### 2.9. Statistical Analysis

All results are expressed as means ± SEM Data were analysed by 1-factor analysis of variance (ANOVA). If a statistically significant effect was found, the Newman-Keuls test was performed to isolate the difference between the groups. *P* values less than 0.05 (*P* < 0.05) were considered to indicate significance.

## 3. Results

### 3.1. Effect of Taurine and LB Extract on Cell Viability in ARPE-19 Cells Cultured in Normal Glucose Condition

**Figure 1:**
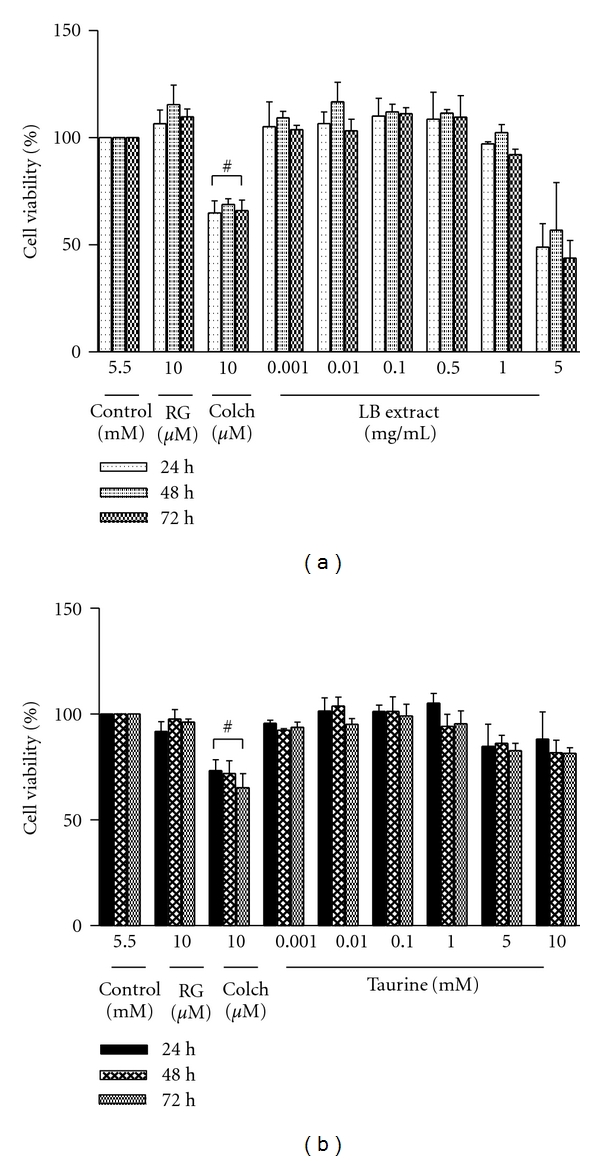
Time- and dose-dependent effect on cell viability following LB extract (a) and taurine (b) treatment. ARPE-19 cells were seeded onto 96-well plates, and LB extract (0.001, 0.01, 0.1, 0.5, 1, and 5 mg/mL) and taurine (0.001, 0.01, 0.1, 5, and 10 mM) were added to the medium for 24, 48, and 72 h. Cell viability was measured by MTS assay. The values of cell viability were normalised to the value of the control. The data represents the mean ± SEM of 5 independent experiments. Rosiglitazone (RG) served as positive control, and colchicine (Colch) served as negative control.

To determine whether LB extract and taurine component influenced viability in normal glucose-treated ARPE-19 cells, MTS assay was performed for 24 h, 48 h, and 72 h of incubation. Incubation of LB extract at 0.001, 0.01, 0.1, 0.5, and 1 mg/mL in normal glucose-treated APRE-19 cells for 24 h and 48 h had little or no cytotoxicity (>90% viability remained). At 5 mg/mL, LB extract was cytotoxic at all incubation times (Figure 1(a)).

Incubation of APRE-19 cells with taurine for 24 h, 48 h, and 72 h had little or no effect on cytotoxicity (>90% viability remaining). At 5 and 10 mM, taurine was slightly cytotoxic (approximately 80% viability remaining) at all incubation times (Figure 1(b)).

### 3.2. Cytoprotective Effects of Taurine and LB Extract in ARPE-19 Cells Exposed to High Glucose

**Figure 2:**
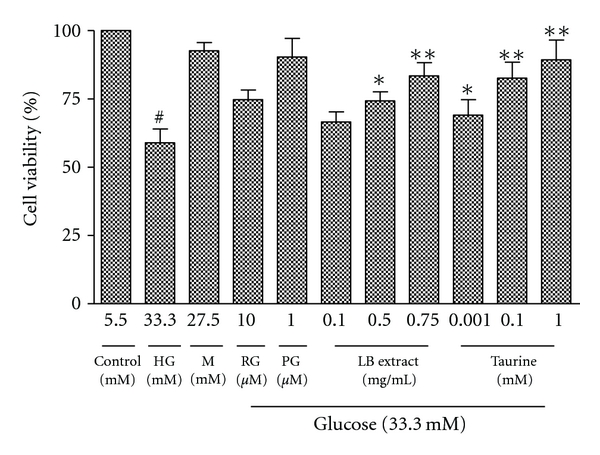
Effect of LB extract and taurine on high-glucose-induced loss of cell viability. ARPE-19 cells pretreated with LB extract (0.1, 0.5, and 0.75 mg/mL) and taurine (0.001, 0.1, and 1 mM) were exposed to high glucose (33.3 mM) for 48 h. Cell viability was determined by MTS assay. Results are expressed as percentage of control and presented as mean ± SEM (*n* = 6 independent experiments). ^#^ *P* < 0.001 versus control; *P < 0.05, ** *P* < 0.001 versus high glucose (HG). Mannitol (M) served as osmotic control.

As it is well established that hyperglycemia induces cell death in retinal epithelial cells [37], ARPE-19 cells were treated with 33.3 mM glucose for 48 h in the presence of LB extract (0.1, 0.5, and 0.75 mg/mL) or taurine (0.001, 0.1, and 1 mM), and their cytoprotective effect was examined by MTS assay. Since high glucose increases osmolarity, ARPE-19 cells were also exposed to an osmotic control (27.5 mM mannitol + 5.5 mM glucose). Cell viability in 33.3 mM glucose was significantly decreased by 59.9%; the loss of viability was reversed by adding LB extract dose dependently (by 67.9, 74.4, and 81.0%, resp.) (Figure 2). Likewise cell viability in 33.3 mM glucose condition was reversed by adding taurine in a dose-dependent manner (69.4, 82.6, and 89.3%, resp.) (Figure 2). Loss of cell viability in high-glucose culture was similarly reversed by adding the positive controls RG (by 73.8%) and PG (by 89.1%). The osmotic control did not show a significant reduction in cell viability.

### 3.3. Effect of Taurine and LB Extract on Cell Apoptosis in High-Glucose-Treated ARPE-19 Cells

To investigate whether the cytoprotective effects of LB extract and taurine were due to the attenuation of high-glucose-induced apoptosis, characteristic morphological staining was performed using Hoechst 33342 and flow cytometry with Annexin V/PI double staining to identify and quantify the apoptotic cells.

**Figure 3:**
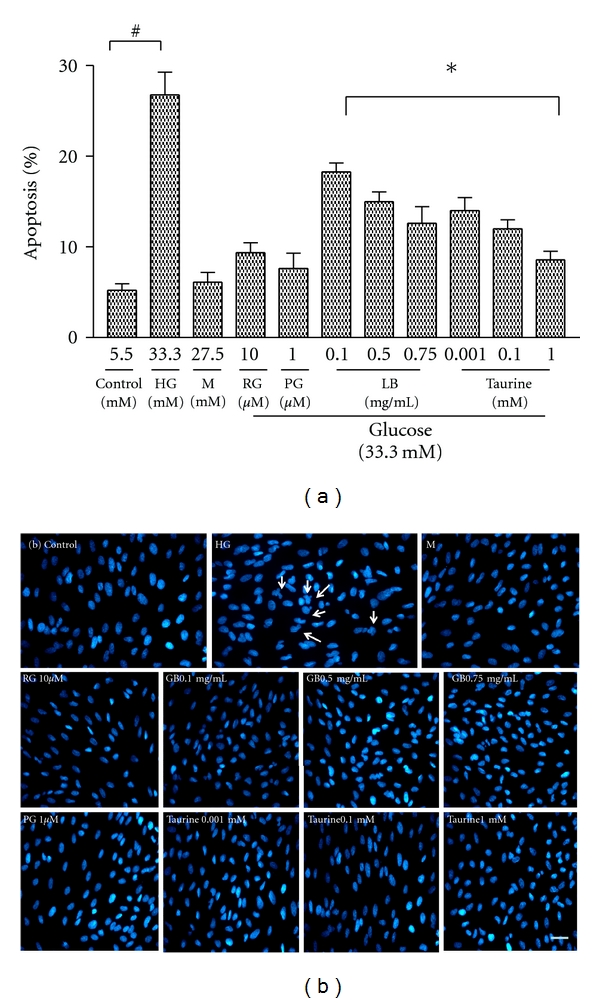
Effect of LB extract and taurine on high-glucose-induced ARPE-19 cells, with condensed and/or fragmented chromatin determined by the fluorescent dye Hoechst 33342 method. (a) Quantification of apoptotic cells in control and in high-glucose-induced ARPE-19 cells treated in the absence and presence of LB extract (0.1, 0.5, and 0.75 mg/mL) or taurine (0.001, 0.1, and 1 mM) for 48 h. Positive controls (RG and PG) and the osmotic control (M) were also used. The results are presented as percentage of total number of cells counted (nonapoptotic + apoptotic) and represent the mean ± SEM (*n* = 6 independent experiments). *P < 0.01 versus HG; ^#^ *P* < 0.05 versus control. (b) Morphological analysis of control (panel 1) and high-glucose-treated ARPE-19 cells with Hoechst staining. Arrows indicate examples of cells with condensed and/or fragmented nuclei. Scale bar 50 *μ*m.

Incubation with high glucose for 48 h induced a significant increase in the number of apoptotic cells (26.8% of total cells), as identified by Hoechst staining, compared with normal glucose culture (Figure 3(a)). The inhibitory effect of LB extract and taurine on high-glucose-induced apoptosis was demonstrated in characteristic morphological staining (Figure 3(b)). Treatment with LB extract (0.1, 0.5, and 0.75 mg/mL) dose dependently decreased the number of apoptotic cells by 18.3, 15.0, and 8.7% of total cells, respectively, compared to the high-glucose control, reaching values comparable to those in the control cultures (5.2% of total cells) (Figure 3(a)). Taurine (0.001, 0.1, and 1 mM) dose dependently decreased the number of apoptotic cells, by 14.0, 12.0, and 8.6% of total cells, respectively, compared to the high-glucose control, approaching values in the control cultures (5.2% of total cells) (Figure 3(a)). The number of apoptotic cells as identified by Hoechst staining in high-glucose condition was similarly decreased by the positive controls RG (9.4%) and PG (7.6%).

**Figure 4:**
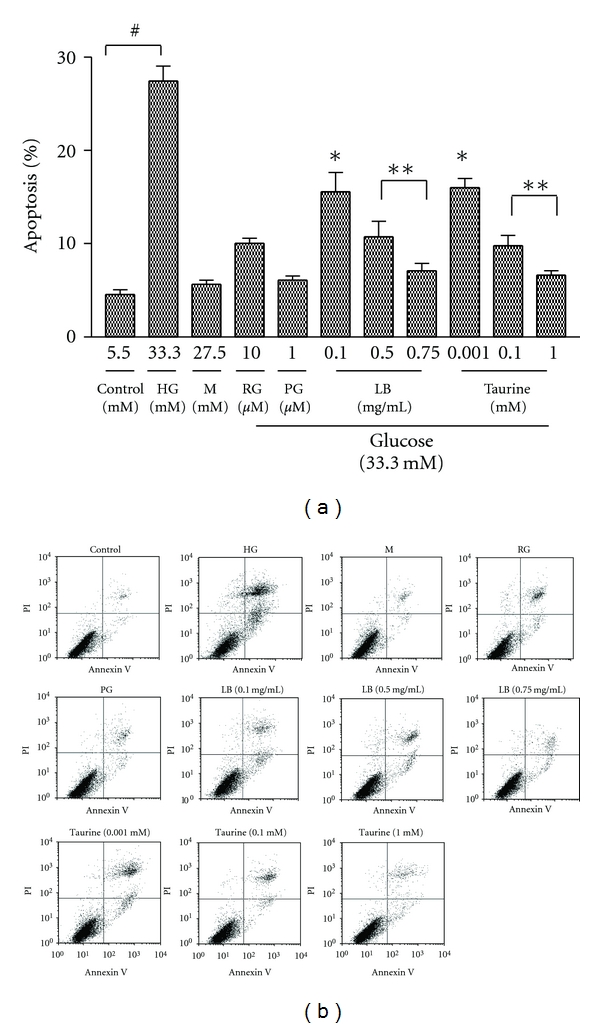
Effect of LB extract and taurine on high-glucose-induced apoptosis of ARPE-19 cells. Cells treated with LB extract (0.1, 0.5, and 0.75 mg/mL) or taurine (0.001, 0.1, and 1 mM) were exposed to high glucose (33.3 mM) for 48 h. (a) Quantification of apoptotic cells (Annexin V-positive) was determined by Annexin V/PI staining detected by flow cytometry. Results are expressed as percentages of Annexin V positive cells compared to control and presented as mean ± SEM (*n* = 6 independent experiments). *P < 0.01 versus HG; **P < 0.001 versus HG; ^#^ *P* < 0.001 versus control. (b) Representative dotplots of control and high-glucose-treated ARPE-19 cells stained with Annexin V and PI.

To further determine whether LB extract and taurine decreased apoptosis in high-glucose-induced ARPE-19 cells, apoptosis was measured as Annexin V binding to positive cells and quantified by flow cytometry, as Annexin V binds specifically to PS, allowing the discrimination between viable and apoptotic cells [38]. Representative dotplots of control and treated high-glucose-induced ARPE-19 cells stained with Annexin V and PI used for quantitation of apoptosis are shown in Figure 4(b). LB extract (0.1, 0.5, and 0.75 mg/mL) dose dependently decreased the number of Annexin V positive cells (14.8, 10.2, and 6.4% of the control) (Figure 4(a)). Taurine dose dependently decreased the number of Annexin V-positive cells (14.8, 9.3, and 6.0% of the control) (Figure 4(a)). The number of apoptotic cells in high-glucose culture was decreased by adding the positive controls, RG (by 10.0%) and PG (6.1%). The osmotic control did not enhance apoptotic cell death, excluding the involvement of osmotic effects of high-glucose concentrations.

### 3.4. Effect of Taurine and LB Extract on Caspase-3 Protein Expression in High-Glucose-Treated ARPE-19 Cells

**Figure 5:**
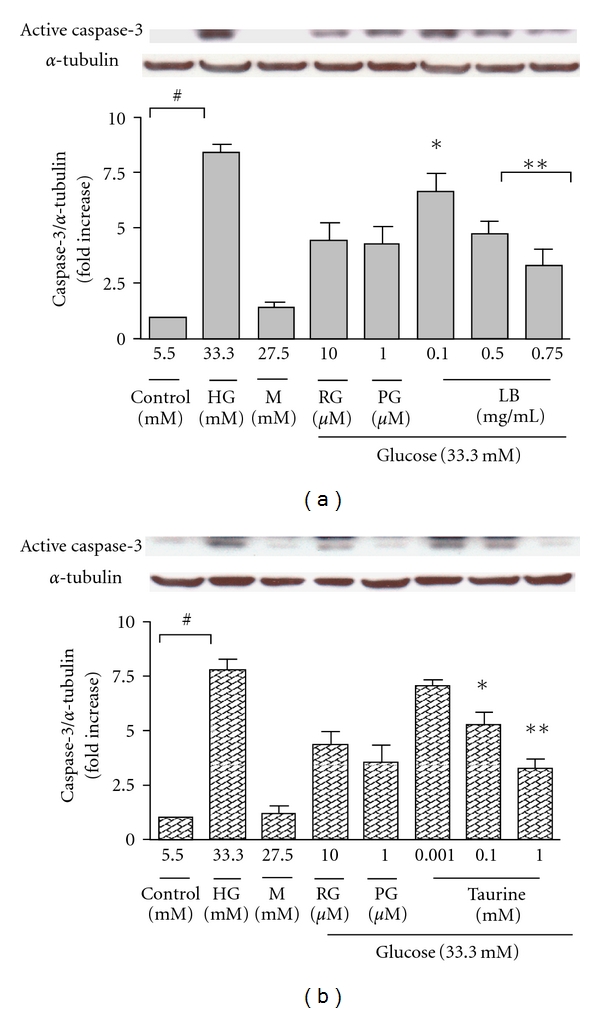
Effects of LB extract (a) and taurine (b) on protein expression of active caspase-3 in high-glucose-treated ARPE-19 cells. The relative level of protein encoding for active caspase-3 was assessed by western blot. Results were normalised to *α*-tubulin. Levels in the control were arbitrarily assigned a value of 1.0. All values are means ± SEM (*n* = 5 independent experiments). ^#^ *P* < 0.001 versus control, *P < 0.05, **P < 0.001 versus HG.

To explore the mechanisms of the anti-apoptotic effect of LB extract, protein levels of active caspase-3 were examined by western blot analysis. LB extract (0.1, 0.5, and 0.75 mg/mL) dose dependently downregulated caspase-3 protein expression (by 6.6-, 4.7-, and 2.7-fold, resp.) (Figure 5(a)).

Taurine (0.001, 0.1, and 1 mM) dose dependently downregulated caspase-3 protein expression (by 7.0-, 5.2-, and 3.3-fold, resp.) (Figure 5(b)).

The protein levels of caspase-3 in high-glucose-treated cells were decreased by adding the positive controls, RG (4.4-fold) and PG (4.3-fold). The osmotic control did not enhance active caspase-3 protein level, excluding the involvement of osmotic effects of high-glucose concentrations.

### 3.5. Effect of Taurine and LB Extract on Caspase-3 Activity in High-Glucose-Induced ARPE-19 Cells

**Figure 6:**
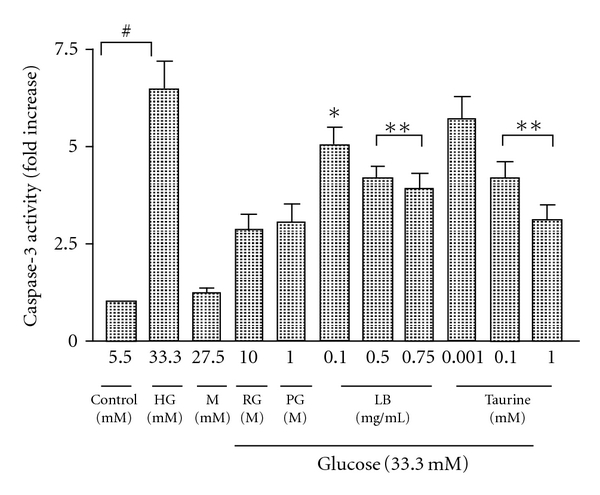
Caspase-3 activity in high-glucose-treated ARPE-19 cells. The effect of LB extract and taurine on fluorometrically determined caspase-3 activity was measured in lysates of high-glucose-treated ARPE-19 cells. The *y-*axis shows the amount of free 7-amino-4-methylcoumarin (AMC) released from the caspase-3-specific substrate DEVD (*N*-acetyl-Asp-Glu-Val-Asp)-AMC. Levels in the control were arbitrarily assigned a value of 1.0. All values are means ± SEM (*n* = 4, each in duplicate). ^#^ *P* < 0.001 versus control, *P < 0.01, **P < 0.001 versus HG.

To confirm the findings of the downregulating effect of LB extract and taurine on apoptosis, activated caspase-3 in apoptotic cells was determined by fluorometric enzyme assay. LB extract (0.1, 0.5, and 0.75 mg/mL) dose dependently downregulated caspase-3 activity (by 5.0-, 4.2-, and 3.9-fold, resp.) (Figure 6).

Taurine (0.001, 0.1, and 1 mM) dose dependently downregulated caspase-3 activity (by 5.7-, 4.2-, and 3.1-fold, resp.) (Figure 6).

The activated caspase-3 levels in high-glucose condition were reversed by adding the positive controls, RG (2.8-fold) and PG (3.1-fold). The increased osmotic control did not enhance active caspase-3 activity, excluding the involvement of osmotic effects of high-glucose concentrations.

## 4. Discussion

Diabetic retinopathy (DR) is a preventable microvascular diabetic complication, and hyperglycemia is considered a major factor in its development [39]. Although various hyperglycemia-induced metabolic abnormalities are implicated in DR [40], it has been difficult to pinpoint the exact pathogenic mechanism, thus making the rationale for a therapeutic target difficult. The results of numerous studies demonstrate that apoptosis is a critical part of the pathology of DR [4, 6]. Apoptotic cell death in retinal regions is a likely stimulus for the increased expression of molecules that enhance the breakdown of the blood retinal barrier (BRB) and lead to vascular proliferation [41]. Indeed, high-glucose-induced apoptotic episodes have been demonstrated by retinal abnormalities, potential visual changes, and the onset of the first vascular change [42].

RPE is essential for neuroretina survival and, consequently, for visual function [43]. In response to damage caused by hyperglycemic condition, RPE cells migrate and proliferate, leading to a breakdown in adhesion between the RPE and the choroidal capillaries, followed by BRB breakdown, compromising blood flow within the RPE layer and leading to eventual retinal edema [37]. In addition, studies have shown that abnormalities in both the structural and secretory functions of RPE cells, followed by photoreceptor apoptosis are found in DR [43, 44].

An objective of the current study was to examine an extract of Goji berry and its major active component on protection of retinal epithelial cell against glucose-induced cytotoxicity as a model of diabetic retinopathy. Diabetes results in various metabolic and biochemical abnormalities in the retina, including increased oxidative stress, which has been shown to induce the expression of the proapoptotic molecules leading to apoptosis. Distinct members of the caspase family are involved in both the initiation and execution phases of apoptosis [45]. Among them, caspase-3 is the “executioner” caspase known to play an important role in the proteolytic cascade during apoptosis [46]. Moreover, the detection of activated caspase-3 is a very reliable tool to identify cells destined to die by apoptosis [47].

Our previous work has established that Gogi berry and its taurine component activate PPAR-*γ* [29]. PPAR-*γ* is heterogeneously expressed in the mammalian eye, prominently present in the retinal pigmented epithelium, photoreceptor outer segments, and choriocapillaris [48]. A recent study has shown that retinal expression of PPAR-*γ* was suppressed in experimental models of diabetes and in endothelial cells treated with high glucose [49]. Moreover, recent studies have suggested that PPAR-*γ* ligands, including 15d-PGJ_2_ (PG) and rosiglitazone (RG), control apoptosis, contributing to tissue protection [50, 51]. Moreover, a screen of FDA-approved compounds identified RG as a novel anti-apoptotic agent in retinal cells both *in vivo *and *in vitro* [52]. Indeed, one recent study has shown that RG protects oxidative stress-induced apoptosis through upregulation of Bcl-2 and modulation of caspase-3 activation [17].

We have investigated the potential of* Lycium barbarum* (LB) as a natural medicine for management of diabetic retinopathy. LB is a traditional Chinese medicine used for centuries in the east and is believed to be beneficial for eye-related pathology [53]. Different biological activities of LB have been demonstrated, including antiaging and cytoprotection [54]. A recent study has shown that the aqueous extract of LB exhibits neuroprotective effects against *β*-amyloid peptide-induced apoptosis in cultured neurons by attenuating the caspase-3-like activity [55]. Moreover, it has been suggested that LB polysaccharides (LBPs) effectively protected the retina from neuronal death and apoptosis by inhibiting proapoptotic signaling pathways, such as c-Jun N-terminal protein kinase (JNK), dsRNA-dependent protein kinase (PKR), and caspase-3 activity in retinal ischemia/reperfusion injury, confirming a neuroprotective role in ocular diseases [56]. In addition, the study showed anti-apoptotic activity of LBP in cultured seminiferous epithelium against hyperthermia-induced damage through the inhibition of superoxide-induced cyt c [57].

In parallel with the studies on LB extract, we have investigated the effect of taurine on apoptotic cytotoxicity pathways in ARPE-19 cells. Taurine is one of the major components in LB present in retina in abundance, where it is essential for sustaining retinal structure and function [25]. Various studies have shown that plasma and tissue levels of taurine are reduced in diabetes [58–60]. Indeed, studies have shown the beneficial effect of taurine supplementation in preventing or ameliorating hyperglycemia-induced retinal defects [58, 59]. Moreover, recent studies have shown that diabetes or high-glucose-induced retinal glial cell apoptosis is inhibited by taurine, exhibiting effective prevention against DR [61]. Anti-apoptotic action of taurine has also been shown to occur by suppressing the Ca^2+^-dependent mitochondrial permeability transition (mPT) to prevent mitochondrial dysfunction and block activation of the cyt c/caspase-3 apoptotic pathway in rat retinal ganglion cells [62]. In addition, taurine exerts neuroprotective effects through an anti-apoptotic effect by increasing Bcl-2 levels, with a decrease in Bax and caspase-3 levels [15].

In previous work which formed the background leading to the present studies, we determined that LB extract and pure taurine, a major component in the LB extract, activated PPAR-*γ* by luciferase reporter gene analysis and by mRNA and western blotting measurement in human retinal pigment epithelial cells. At the same time, both LB extract and pure taurine inhibited a variety of PPAR-*γ*-dependent downstream effectors in the retinal cells [29]. In the present study, we hypothesised that taurine and the LB extract may have a modulating effect on high-glucose-induced apoptosis through PPAR-*γ*-mediated caspase-3 pathway, responsible for their effects in DR. In order to test this hypothesis, we first investigated the effect of taurine and LB extract on modulating cell viability and therefore apoptosis in high-glucose-treated ARPE-19 cells. Our results demonstrated that a methanol extract of LB dose dependently overcame the decreased cell viability in high-glucose-treated ARPE-19 cells (Figure 2). Taurine had a similar effect on overcoming decreased cell viability (Figure 2). The cytoprotective effect of taurine was associated with the attenuation of high-glucose-induced apoptosis, which was shown by characteristic morphological staining (Figure 3(b)) and Annexin V/PI double staining (Figure 4(b)), and a dose dependent decrease in the number of apoptotic cells (Figures 3(a) and 4(a)). Moreover, the results have shown that taurine and the LB extract dose-dependently downregulated caspase-3 protein expression (Figures 5(a) and 5(b)) and inhibited the enzymatic activity of caspase-3 (Figure 6). Therefore, the cytoprotective effect of LB extract parallels the profound suppression of high-glucose-induced apoptosis at the site of caspase-3 regulation. The effects of taurine closely mimicked the effects of the methanolic LB extract, since they occurred at concentrations 0.001–1.00 mM which we showed previously [29] were present in the range of methanolic extract used in the treatments (0.1 to 0.75 mg/mL). However, we cannot exclude the possibility that other components may also contribute to the cytoprotective effects of LB.

In summary, this study has demonstrated for the first time that the traditional Chinese medicine *Lycium barbarum *is cytoprotective against high-glucose cytotoxicity in retinal pigment epithelial cells, at least in part by regulating apoptosis as a result of caspase-3 modulation. This may occur through the initial PPAR-*γ* activation found in previous studies [29]. The effects of the extract are closely mimicked by taurine at concentrations present in the extracts. This pathway and demonstration of its major active component provide a rationale for the therapeutic use of taurine and the valuable medicinal herb LB for the prevention of DR. However, further investigation into their specific mechanisms is warranted to gather proof of efficacy and safety of taurine and LB for protection against DR in various preclinical and clinical settings.
